# Unraveling Complexity: A Case of Possible Overlapping Between Mixed Connective Tissue Disease and Systemic Lupus Erythematosus With Renal Involvement

**DOI:** 10.7759/cureus.60839

**Published:** 2024-05-22

**Authors:** Dwight Smith, Devaun M Reid, Abraham A Mascio, Britannia O Noel, Martin Giangreco

**Affiliations:** 1 Internal Medicine, University of South Florida Morsani College of Medicine, Tampa, USA

**Keywords:** sle and lupus nephritis, focal segmental glomerular sclerosis, autoimmune, systemic lupus erythema, mixed connective tissue disorder

## Abstract

Autoimmune diseases, a term encompassing conditions where the immune system targets its own cells, consist of various pathologies, two of which are systemic lupus erythematosus (SLE) and mixed connective tissue disorder (MCTD). We present the unique case of an anti-ribonucleoprotein (RNP)-positive patient exhibiting renal pathology consistent with lupus nephritis and an additional collapsing variant of focal segmental glomerulonephropathy, who initially presented to the emergency department with signs and symptoms of pneumonia and portal vein thrombosis that were subsequently treated. Conflicting accounts of her autoimmune history led to an extensive workup during her stay, which yielded a tentative diagnosis of SLE vs. MCTD during her current hospitalization for pneumonia. The diagnostic labs revealed conflicting serological markers, with delayed anti-Smith positive results favoring lupus due to its high specificity. A subsequent renal biopsy showed complex renal involvement, suggesting SLE, despite initial positive anti-RNP antibodies known to be protective against renal pathology and classic for MCTD. Complicating matters further, the renal biopsy findings extended beyond common SLE pathology, including additional focal segmental glomerulonephritis (FSGS) involvement. Despite this uncertainty, the patient was treated as if solely having SLE, and immunosuppressives that could have been utilized for the possible MCTD component were avoided due to minimal signs of inflammation/immune response and normal kidney function. This case highlights the difficulty in accurately classifying lupus and MCTD, emphasizing the need for precise diagnosis for tailored patient care. Ongoing research is crucial to refine diagnostic criteria and improve patient outcomes.

## Introduction

Systemic lupus erythematosus (SLE) is an autoimmune disease that manifests with varying degrees of diverse organ involvement, primarily affecting minority women [1]. Among the many clinical presentations, approximately 50% of SLE patients present with renal disease related to lupus nephritis, which has serious comorbidity implications and should be identified and treated early to avoid end-organ failure [1]. Clinically, a patient often presents with symptoms of fever and arthralgia [1]. The cutaneous manifestation of the malar rash is especially telling [1]. Subsequent laboratory tests for serological biomarkers associated with SLE include antigen-specific antinuclear antibody (ANA), with positive results prompting additional testing targeting double-stranded DNA (dsDNA) or ribonucleoprotein (RNP) complexes (Ro/SSA, La/SSB, Smith, and RNP) [1].

Identifying potential renal involvement in SLE relies on an initial urinalysis and measurement of kidney function via serum creatinine concentration, or estimated glomerular filtration rate (EGFR), highlighting the presence of proteinuria and hematuria, regardless of the presence or absence of flank pain [2]. Proteinuria ≥500 mg/day necessitates a follow-up kidney biopsy to define the nature of possible renal pathology [2]. Lupus nephritis usually presents as an immune-complex-mediated glomerulonephritis involving immune complex deposition in the kidneys and other organs [2]. SLE treatment typically involves the use of non-targeted immunosuppressives such as mycophenolate mofetil (MMF) or high-dose corticosteroids plus cyclophosphamide [2].

Mixed connective tissue disease (MCTD) is a less understood autoimmune condition that often shares overlapping characteristics with SLE, such as Raynaud’s phenomenon [3]. Anti-U1RNP is described in the literature as the primary characteristic marker of MCTD, although its lack of specificity is noted, as levels are not always high in all MCTD patients [4]. In this case, anti-RNP antibodies were observed to be protective against severe renal involvement, particularly diffuse proliferative glomerulonephritis, representing a process often mediated by immune-complex deposition [5]. As a result, MCTD patients typically possess antibodies protective against renal pathology, further shielding against immune-complex-mediated processes commonly seen in SLE, providing a diagnostic contrast point for physicians to distinguish between MCTD and lupus etiology.

## Case presentation

A 29-year-old African American female with a complex medical history, including an uncertain diagnosis of either SLE or MCTD, antiphospholipid syndrome, obesity, and a history of gastric sleeve surgery, presented to the emergency department (ED) with a range of symptoms, including nausea, vomiting, fevers, chills, and exertional dyspnea. These symptoms had initially manifested a couple of months earlier when she sought care at a local hospital due to weakness and dizziness. At that time, a portal vein thrombosis was identified, and she was started on 20 mg of rivaroxaban. Given her history of antiphospholipid syndrome, she was subsequently referred to rheumatology, where she received a diagnosis of MCTD and an outpatient referral to oncology for chest lymphadenopathy. The workup for the MCTD diagnosis was not available in the charts.

Approximately one week before her admission to the ED, she was admitted to the hospital due to dyspnea, nausea, and fevers and was diagnosed with pneumonia. After receiving two days of 4.5 g intravenous piperacillin/tazobactam, she was discharged without antibiotics but was transitioned to 5 mg warfarin with a subcutaneous 80 mg enoxaparin bridge due to a symptomatic increase in the size of her suspected portal vein thrombosis despite adherence to rivaroxaban. However, her symptoms persisted, prompting her presentation to Tampa General Hospital (TGH) on the day of admission. She reported vomiting food she had consumed earlier that morning, with no accompanying diarrhea or abdominal pain. Notably, she had lost approximately 60 lb since her gastric sleeve procedure approximately two years prior. The patient was a G3P1021 with one stillborn birth and one elective abortion in her obstetric history. Strikingly, the patient's autoimmune disease history was inconsistent, as she reported being diagnosed with lupus and antiphospholipid syndrome during a previous pregnancy but was recently informed during a rheumatology visit that she had MCTD instead of lupus. This discrepancy resulted in a significant portion of the patient's hospital stay being devoted to determining whether she had lupus or MCTD, with her condition being tentatively labeled as SLE vs. MCTD throughout her 17 days of admission.

**Figure 1:**
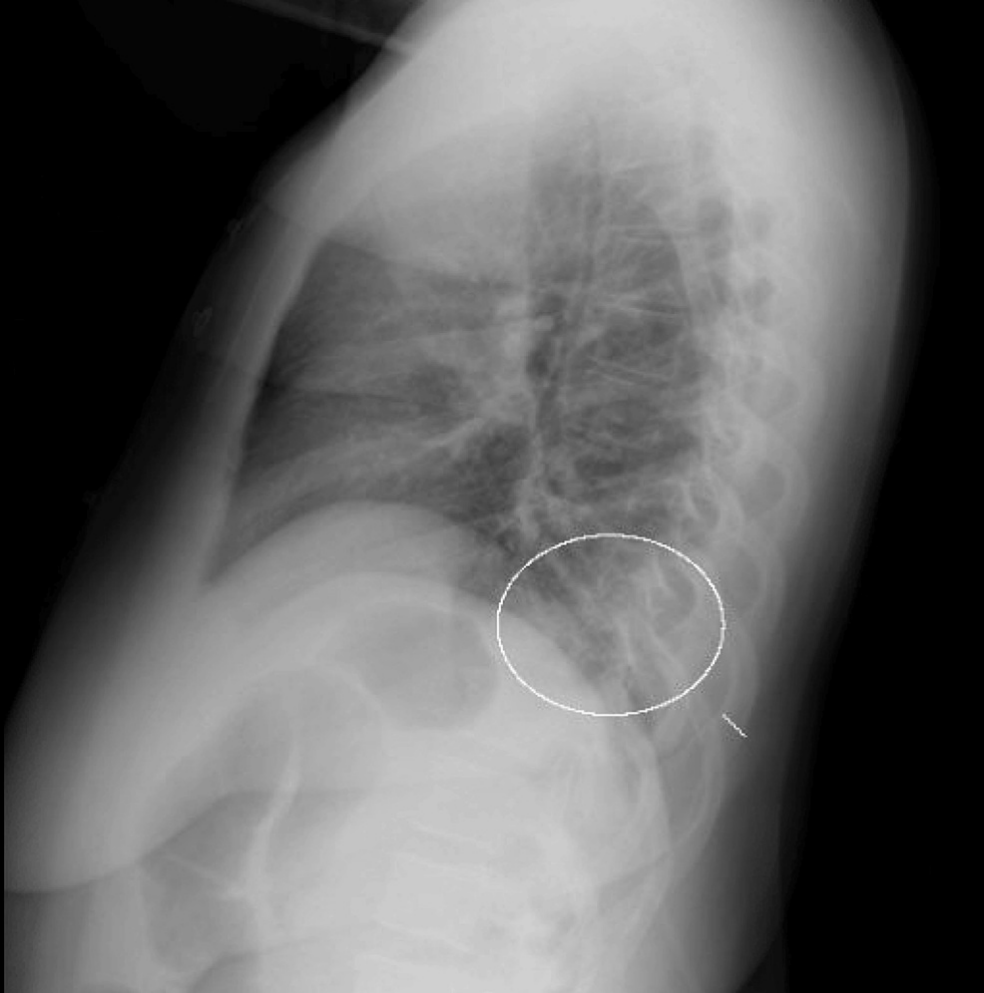
The white circle denotes posterior right lower lobe airspace disease overlapping the right hemidiaphragm. The radiographic findings are suspicious for right lower lobe pneumonia.

**Figure 2:**
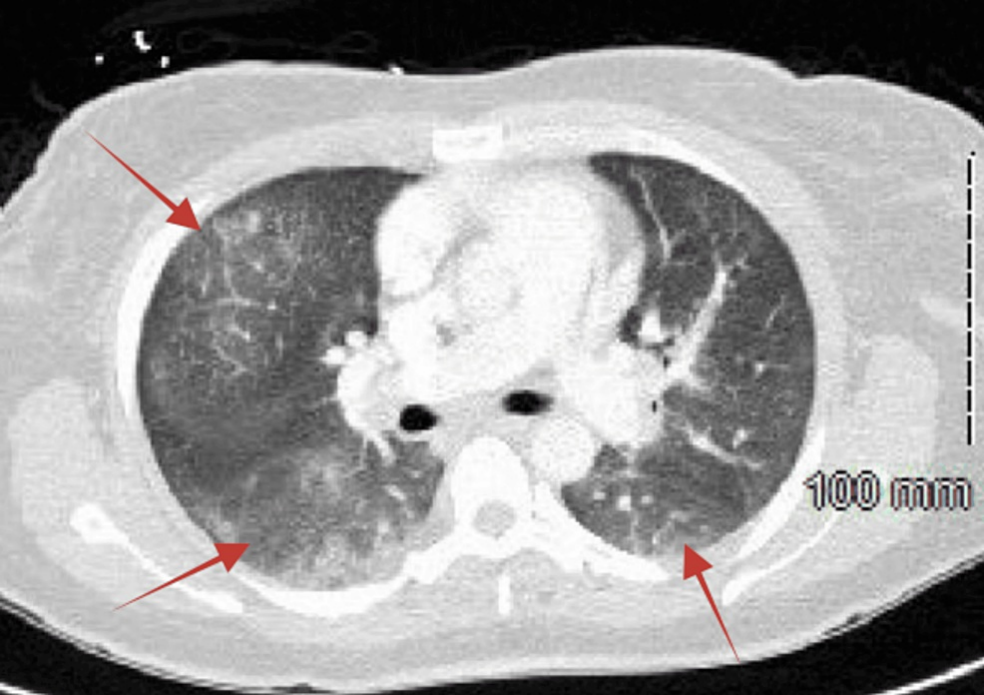
The chest CT shows multifocal bilateral patchy opacities (arrows), predominantly involving the lower lobes and right middle lobe.

Upon physical examination, the patient was found to be febrile at 103.4 °F (39.7 °C) with a pulse of 88 beats per minute, and light rhonchi were auscultated at the base of the right lung. Laboratory tests revealed several abnormalities, including low hemoglobin at 10.4 g/dL, low hematocrit at 34.5%, low platelets at 99 x 10^3^/µL, low sodium at 129 mmol/L, low calcium at 8.2 mg/dL, low albumin at 2.4 g/dL, and elevated aspartate aminotransferase at 98 U/L. Subsequent imaging studies, including a chest X-ray (Figure 1) and CT scan (Figure 2), revealed features consistent with multifocal pneumonia, which was subsequently treated with broad-spectrum antibiotics, including intravenous vancomycin and piperacillin/tazobactam. Given her history of potential liver clot, a Doppler ultrasound of the liver was performed, revealing a portal vein thrombosis, which was subsequently treated with anticoagulants, including warfarin and enoxaparin.

Regarding the investigation into SLE vs. MCTD, an initial rheumatology consultation identified a constellation of findings from the patient's medical history, along with laboratory serological markers suggestive of MCTD, including Raynaud's phenomenon, lymphopenia, possible interstitial lung disease, high-titer positive RNP, and high-titer ANA speckled 1:1280. However, positive SSA/SSB (negative SICCA) was also present, suggesting SLE. Rheumatologists also noted a clinical diagnosis of seronegative antiphospholipid syndrome related to stillbirth and current portal vein thrombosis. Ultimately, delayed labs showing high anti-Smith markers led the doctors to favor lupus over MCTD as the underlying rheumatologic etiology.

**Figure 3:**
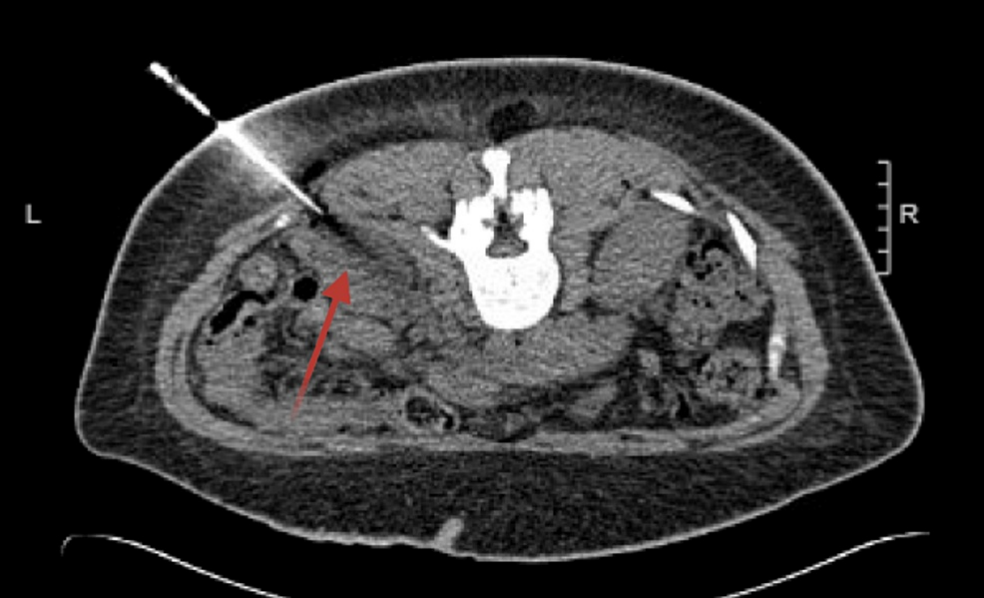
Technically successful CT-guided native left renal biopsy (arrows pointing to the site of the biopsy).

Nephrotic proteinuria was also identified, prompting nephrology to advocate for a renal biopsy, despite the patient testing positive for the seromarker found to be protective against renal involvement, anti-RNP. Upon biopsy (Figure 3), unusual renal involvement was found in the patient, consisting of features of a collapsing glomerulonephropathy variant of FSGS and early membranous nephropathy, likely in the setting of SLE (class V). The patient's continuous infusion of heparin was temporarily halted for a few days post-procedure due to an increased risk of bleeding.

Physicians chose not to initiate immunosuppressive therapy due to the patient's stable renal function and low-grade proteinuria of only 0.26 g/g, as they suspected that the initial nephrotic range proteinuria may have been an overestimation due to contamination from menstrual blood. The patient was subsequently discharged with enoxaparin skin injections and warfarin, feeling well and asymptomatic, and later began treatment with hydroxychloroquine and mycophenolate tablets for SLE. Since then, the patient has been readmitted to the ED for various causes of dyspnea, including community-acquired pneumonia and sepsis, which were subsequently treated.

## Discussion

The presented case delves into the complexities surrounding the differentiation between SLE and MCTD, two chronic autoimmune diseases characterized by overlapping pathologies and clinical features. Our patient's case challenges conventional diagnostic paradigms, highlighting the need for refined diagnostic criteria and therapeutic approaches in such cases.

In the routine workup of patients suspected of autoimmune diseases, clinicians often rely on serologic markers to guide diagnosis. However, our case presented a unique challenge with conflicting serological markers, including anti-RNP, typically associated with MCTD, and anti-Smith, associated with SLE. Despite the eventual emergence of anti-Smith positivity, initially leaning toward an SLE diagnosis, the presence of anti-RNP added complexity to the diagnostic process.

However, our patient exhibited a complex renal pathology, including features consistent with SLE, such as class V lupus nephritis, alongside an uncommon collapsing variant of focal segmental glomerulosclerosis (FSGS), which typically necessitates aggressive treatment with glucocorticoids and immunosuppressives [6]. This variant may have implications for treatment response, particularly in African American patients with APOL1 risk alleles [7]. However, the physicians in our case opted against aggressive intervention due to the patient's apparent normal kidney function and low-grade proteinuria, emphasizing the importance of exercising caution and conservatism in managing renal pathology.

Following initial treatment for pneumonia and liver clots, subsequent management primarily focused on lupus-related therapies, possibly driven by the perceived risk of lupus nephritis-related morbidities, noting that such a demographic has a much lower 20-year survival than SLE patients without renal pathology [8]. The ongoing presence of anti-RNP positivity added further complexity to the diagnostic process, emphasizing the challenges of delineating between SLE and MCTD based on serological markers alone.

Efforts to refine diagnostic criteria and distinguish between SLE and MCTD are underway, with studies exploring novel molecular markers and classification criteria showing promise for improving diagnostic accuracy [9]. However, the lack of established guidelines and ongoing research highlights the need for further investigation into diagnostic methodologies and clinical presentations to enhance our understanding and management of these complex autoimmune diseases. In conclusion, our patient's case highlights the intricacies involved in distinguishing between SLE and MCTD, highlighting the limitations of current diagnostic criteria and the need for more nuanced approaches to diagnosis and treatment in such cases.

## Conclusions

This case of a 29-year-old African American woman, initially presenting with pneumonia symptoms, underscores the diagnostic and therapeutic challenges in distinguishing between SLE and MCTD. The complexities arise from conflicting serological markers and the necessity of integrating clinical findings with laboratory data for accurate diagnosis. Key aspects include the identification of complex renal pathology, such as class V lupus nephritis combined with a rare variant of FSGS, necessitating tailored treatment strategies. Despite serological evidence suggesting MCTD, the predominance of lupus-related therapies highlights the difficulty in distinguishing between these autoimmune diseases and emphasizes the importance of individualized patient care. This case not only stresses the critical role of specific investigations, such as renal biopsy, in guiding treatment but also signals the ongoing need for research to refine diagnostic criteria and therapeutic approaches to enhance outcomes in autoimmune conditions.
